# Translation of the shortened dental arch research into clinical practice: a stakeholder mapping approach

**DOI:** 10.1038/s41405-020-0039-3

**Published:** 2020-07-28

**Authors:** Saadika B. Khan

**Affiliations:** Department of Restorative Dentistry, Faculty of Dentistry, University of the Western Cape, Cape Town, South Africa

**Keywords:** Dentistry, Removable prosthodontics

## Abstract

**Aim:**

To identify key participants that can ensure implementation of the SDA or PRDA as a prosthodontic management option using a stakeholder mapping approach.

**Methods:**

A stakeholder mapping approach is employed which is a strategic method to identify, rate the importance of input and the influence, highlighting how clinical implementation can be ensured. A stakeholder map was used as the research tool. Stakeholders were classified according to their level of influence in either assisting with change or obstructing progress as well as the impact of their input within the dental organization and the broader South African environment.

**Results:**

Several stakeholders were identified and were classified in two ways: Primary or secondary and according to their affiliation with the organization where change needs to occur. Initially, a lecture on the shortened dental arch was included in 4th year of undergraduate study, after consultation with the head of the department. This was abandoned as students misunderstood the use of the concept related to clinical requirements; thus, the location of where this concept must be taught, was reconsidered. The role of other key stakeholders that could effect change was also highlighted with this approach.

**Conclusion:**

This strategic analysis allowed identification of key stakeholders and their roles that can assist with implementation of the SDA or PRDA, some of whom should be addressed further to ensure alignment of practices to health policies.

**Key points:**

1. Knowledge translation consists of multiple stages from design to implementation which includes diffusion, dissemination (such as publishing) and implementation of evidence into clinical practice (application of concepts or procedures to improve patient care).
2. Only quality research, as stipulated on the evidence pyramid, can be used to change curricula and clinical practices.
3. The strategic approach with stakeholder mapping allows identification of key stakeholders in prosthodontics (knowledge brokers or communities of practice) that have the interest and influence to change curricula and clinical practice; including a combined approach with researchers which may enable easier application of quality care to patients.

## Introduction

The classic shortened dental arch (SDA) as a prosthodontic concept was originally described by Käyser and comprises of 20 occluding anterior and premolar teeth only.^1,2^ This SDA treatment option, now also referred to as a posteriorly reduced dental arch (PRDA) due to the different combinations of missing posterior teeth, may be considered as a beneficial treatment approach for developing countries such as South Africa (SA).^3–6^ Recognition of the SDA concept by the World Health Organization (WHO) in 1982 stipulating that “the retention of 20 anterior and posterior teeth for young adults suffice for adequate functioning,” allowed the SA government to adopt it into policy since 1994 when reviewing all health policies to include evidence-based concepts.^7–9^ But at the time of its inclusion into policy, no contextual evidence that can attest to its benefits for the SA population, were available.^3–6^ Subsequently more global, and now contextual evidence has indorsed its use but to date its absence in clinical practice is rather conspicuous and the teachings highlighting its inclusion in treatment planning is also minimal.^1–6,10–22^ Following the research conducted in the SA context, the need to address the absence of the SDA or PRDA in both the curriculum and in clinical practice which would optimize and enable the delivery of quality evidence-based concepts became significant.^3–6^ This paper therefore attempts to describe the stages following research diffusion and active dissemination of evidence within the academic community which occurs at conferences and via publications. A combined approach to assist with implementation of the SDA or PRDA concept clinically is thus emphasized including other aspects of knowledge sharing with non-academic stakeholders and how to actively engage them to enable delivery of improved evidence-based care to patients.

### SDA South African evidence

Globally, researchers have conducted studies related to the SDA concept clinically but generalizability of these results to communities that are vastly different are always of concern.^2,10–22^ The teachings at all SA dental schools largely focus on the traditional model, namely one that conforms to the conventional principle of the need for restoring and extending dental arches to include 28-teeth.^3,4^ In a SA dental school where the SDA research was conducted, it was found that the information related to the SDA was only mentioned when discussing the management of mandibular bilateral distal extension scenarios.^4^ Subsequent teachings were, however, modified to include information on the SDA in the 4th year of the dental program, but students became confused when to implement it as other modules and the compulsory clinical requirements for students were not aligned to this thinking.^4^

The healthcare policies, including WHO and African Regional Oral Health Strategies 2025 and the current National Health Strategy Goals 2030 of SA, recommend that a primary healthcare and evidence-based approach be adopted in health management.^7,9^ The need for evidence-based dentistry (EBD) is significant as it guides academics and practitioners when revising and improving teaching material and treatment options for patients.^23–28^ This type of reflective approach and planning is expected in healthcare settings as it relates to the developments in dental technology, materials and updating of clinical procedures.^23–28^

But evidence can only be translated into clinical practice depending on the type of research and the rigor in the design of the study.^29,30^ For example, a clinical study where a randomized controlled trial (RCT) design was used is more reliable and valid and these results may easily translate into practice, compared with a laboratory or a questionnaire study.^29,30^ Research completed in the southern part of SA were from the apex of the evidence pyramid, for example, a systematic review and a RCT.^3–5,29–32^ Suffice it to say, substantial evidence is consequently available that confirm the functionally-effective advantages of using the SDA or PRDA as an evidence-based alternative treatment option.^1–6,10–22^ This evidence may also be used to update the teachings and clinical practices, following reflection of the curriculum and its contents.^1–6,10–22^ Thus the provision of expensive prosthodontic mechanical interventions could be minimized, especially amongst underprivileged societies.^4–6^

**Fig. 1:**
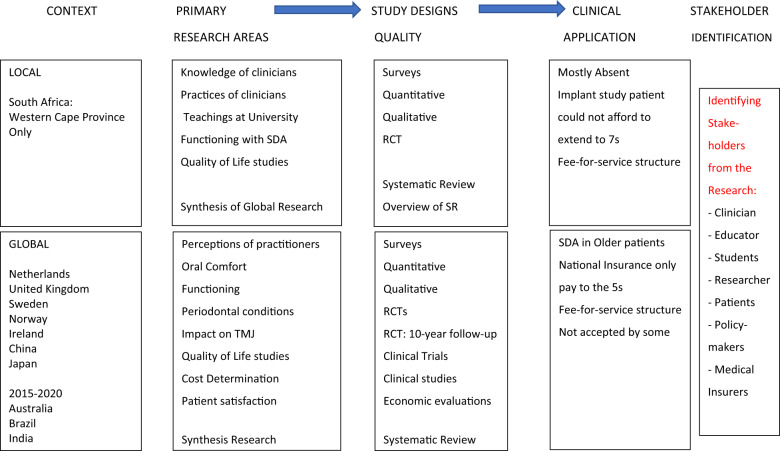
Evidence for the shortened or posterior reduced dental arch.

Moreover, this SDA or PRDA treatment approach is considered valuable especially for the historically-disadvantaged and rurally-based communities of SA where accessibility to treatment is limited.^1–6^ The evidence from the SA perspective included understanding key SDA- or PRDA-related areas, determining knowledge of professionals (clinicians and students) and exploring the oral functional level, patient satisfaction and oral health-related quality of life (OHRQoL) using high-end clinical and synthesis research.^3–5,31,32^ Thus significant participants were included for the different areas of SDA research, namely the educators (both classroom and clinical), dental students, clinicians (general dentists and specialists) and partially dentate patients with varied PRDAs (Fig. 1).^3–5,31^ But, after having been exposed to a traditional teaching model, it is much more challenging to change set clinical practices after graduation, even though continuous professional development has become compulsory.^3,4^ To assist in changing this kind of mindset, translating high-quality research into practice can be accomplished by adopting a stepwise approach to clinical implementation with a combined strategy to improve delivery of evidence-based care to patients.^33,34^

### Knowledge translation

Knowledge translation (KT) refers to the assessment, review and utilization of scientific research (evidence) to improve the conditions of patients, where appropriate.^33–39^ From knowledge-into-action or from evidence to practice, entails the translation of best evidence obtained from rigorous quality research methodologies into clinical practice to improve patient care.^33–40^ The KT process thus consists of multiple stages from design to implementation including diffusion, dissemination (such as publishing and conference presentations), and implementation of evidence.^33–40^ Diffusion is largely a passive stage mediated by peers, whereas the dissemination part of KT can be passive (shared with academics and researchers) or active (where specific audiences are targeted) with the implementation phase being rather active (which include a systematic effort to adopt research outcomes).^33–38^ The focus of this paper will therefore be on the active part of dissemination and largely on the implementation phase, identifying the strategies required, those that may hinder this process and how to overcome any barriers.^33–40^ This active phase of dissemination is, however, not a linear chronological process as several of these stakeholders, some of whom were identified following completed SDA or PRDA research, may be addressed at the same time, over a period of time or only when possible (Fig. 1).^40–42^ Having identified key stakeholders is only one aspect of addressing the implementation phase, the difficult aspect is knowing how and when to, and by whom these participants will be addressed that could impact their actions to make a difference.^40–42^

For the practical implementation of the research, several tools, frameworks, and techniques are available and choosing the correct one is a process in itself.^39^ it is crucial that the framework speaks to key role players who will be instrumental in the implementation of the evidence as the ultimate goal is to change clinical behavior that will benefit patients while receiving quality care.^39^ Frameworks such as the “Knowledge to Action” cycle or the “Promotion Action on Research Implementation in Health Services (PARIHS)” or the value co-creation models can easily be followed, though for this paper stakeholder mapping was employed.^39–42^ Stakeholder engagement in research is defined as an iterative process of actively soliciting the knowledge, experience, judgment, and values of individuals (academic and non-academic) selected to represent a broad range of interests in a particular issue, for the dual purposes of creating a shared understanding and making relevant, transparent and effective decisions.^39–42^

The details of stakeholder mapping methodology are described in the sections below and according to these specifics, there are clear advantages to using this approach.^40–43^ It allows identification of influential stakeholders in the field, who could ensure implementation of the SDA or PRDA concept in clinical practice.^39–44^ Once their support or influence is guaranteed, the less significant participants would respond instantaneously, making changes to practices so much easier.^39–44^ Knowing these influential stakeholders and keeping the communications frequent, allows them to gauge the significance of changing clinical protocols as relates to the SDA or PRDA.^39–44^ There are, however, also limitations to this analysis, such as over- or under-estimating the influences or incorrect prioritization of key stakeholders, or excluding some from important communications by not trusting them.^39–44^

The rationale for the stakeholder mapping approach, is based on knowing that evidence-based practice, which is an accepted method of updating knowledge and direct educational interventions, is found to not be very effective in influencing clinical behaviors and practices.^39–44^ Thus, completed quality research, even high-end studies, does not necessarily and automatically translate into clinical practice. In SA, following contextual research related to the SDA or PRDA, the dissemination of this evidence has mostly occurred in the form of publications and presentations at both national and international conferences (the passive aspect of dissemination of KT).^3–5,31^

Attempts have been made to improve on teachings within the educational sector, but this has been rather limited. Following the SDA or PRDA research and publications, the researcher’s expectation was that the clinicians would, after having read and reflected upon the SDA or PRDA concept, attempt to change clinical practices accordingly, but this was not found to be the case. The purpose for completing this stakeholder mapping is therefore based on wanting to start communicating directly with clinical practitioners and other important stakeholders that could influence the decisions where patients are offered this SDA or PRDA treatment option.

## Aim

To identify key participants whose influence may impact or even ensure implementation of the SDA or PRDA as a prosthodontic management option using a stakeholder mapping approach.

## Objectives

The objectives required to achieve the aims of this study include:

1. To identify the different stakeholders that could play a meaningful role in implementing the SDA or PRDA treatment option.
2. To group the different stakeholders into key roles that may guide this process of active dissemination of KT which could affect a change in clinical practice.
3. To elaborate on the communication plan for each stakeholder that will serve as a guide in this process of implementation.

## Methods

The methodology utilized is stakeholder mapping or analysis, where key participants are identified that can influence the translation and implementation of the SDA or PRDA evidence into clinical practice.^39–44^ Engagement and reporting of any communications with stakeholders follow the guidelines issued by the Declaration of Helsinki obtained with studies registered at the time of conducting it.^45^ Thus, subsequent to the research completed no attempts were made to contact participants, that could affect the ethics obtained.^45^ To address the active phase of dissemination of KT identified as lacking above, this stakeholder approach seemed appropriate, after identifying some of the key participants from the completed research (Fig. 1).^3–5,31,33–44^

The steps or methodology for this stakeholder mapping process include:^39–44^

(i) Identifying or listing key stakeholders as they relate to the SDA or PRDA concept within the field of prosthodontics; that is all those involved with the teaching, constructing, implementing (clinically or policy), and remuneration of procedures and those receiving treatment (patients).
(ii) Prioritizing stakeholders according to their level of influence regarding the SDA or PRDA in the different academic and non-academic environments such as the teaching setting, impact on patient treatment and implementation in clinical practice and policy-making conditions.
(iii) Identifying the communication plan: who (participants), when, by whom and how (medium by which) the specific stakeholders identified related to the SDA or PRDA could be addressed.^39–44^

The steps for this mapping approach outlined above will be repeated within the results section as it assisted the researcher in describing what was achieved and what still needs to be completed. As with other research methodologies, this mapping approach does not adopt a sequential linear manner as outlined above, but several of the stakeholders may be addressed at the same time or after a waiting period (especially when evaluating the reactions of one participant or once an aspect thereof was completed).^39–44^

The research tool used for this process is called a stakeholder map or a stakeholder communication plan and this was used for this aspect of the SDA or PRDA research.^39–42^ By implementing a stakeholder mapping approach, the researcher was ensured of being guided with the active phase of dissemination in the KT process in a stepwise manner.^33–42^ The stakeholder communication plan, which is the most important part of this approach, is described in detail in the results section according the greatest impact it may have on the particular participants while addressing the objectives of this study.^33–42^ A narrative analysis and synthesis of collected information was completed.

## Results

The results of this stakeholder mapping approach are recorded on (Table 1). The results are reported as outlined by the 3-step approach in the “Methods” section and the objectives of the study.^33–42^ The strategic approach to identify, rate importance of input and influence (creating a matrix of power and interest) to ensure implementation of the SDA concept was also completed.^39–44^

**Table 1:** Stakeholder analysis: mapping of stakeholders relating to the management of the SDA or PRDA implementation.

Stakeholder	Purpose of engagement	Key interest & main message/issues	Communication/forum/vehicle	Frequency	Comments
A: Academics					
1. Head of Prosthetics	Improve teachings	Evidence-based teachings and practices *Curriculum change* Quota inclusion	Face-to-face; Departmental meeting SELF	Lecture inclusion in 5th year	Include clinical teachers Shared evidence Tried in 4th year module
2. Prosthodontists	Improve teachings	Evidence-based teachings and practices *Curriculum change* Quota inclusion	Face-to-face; Departmental meeting SELF	Seminar per year	Include clinical teachers Shared evidence Part of SDA research
3. Researchers	Evidence	Quality research/evidence *Implementation*	Face-to-face; Conferences; workshop SELF	Research meetings annually	Include academics, practitioners, organizations Shared evidence Conference presentations
4. Dental technicians Dental auxiliaries	Inform teachings	Evidence-based teachings and practices	Face-to-face; Departmental meeting SELF/Head of Prosthodontics	Quarterly meetings	Include colleagues Shared evidence
B: Policymakers					
1. Government	Effect policy Public action	Policy implementation Awareness programs	E-mail Face-to-face meetings Infographics PROFESSOR/SELF	Before policy changes	Provide evidence to assist with implementation Aligned to WHO policy
2. Oral health insurers	Evidence awareness Action	Policy implementation Awareness programs	E-mail Face-to-face meetings Infographics PROFESSOR/SELF	Year-end as insurers modify rules	Provide evidence to effect change in policy and practice Aligned to WHO and SA Oral Health Policy
C: Practitioners					
1. General Dentists	Evidence awareness Effect practice	Evidence-based practice Treatment option Cost saving	Professional meetings (CPD) Conferences: posters, articles Social media: Twitter, Instagram, Facebook, LinkedIn PROFESSOR/SELF	Frequent reminders	Included colleagues Shared evidence Conferences and Group meetings, Part of SDA Research
2. Prosthodontists	Evidence awareness Effect practice	Evidence-based practice Treatment option Cost saving	Professional meetings (CPD) Conferences: posters, articles Social media: Twitter, Instagram, Facebook, LinkedIn PROFESSOR/SELF	Frequent reminders	Included colleagues Shared evidence Conferences and Group meetings, Part of SDA research
D: Patients					
1. General public	Awareness, Educate, Rights	Treatment option Costs Policy inclusion	Media (print, online); Social media sites Posters, billboards SELF, General practitioners	Frequent reminders	Include community leaders to inform of evidence Champion
2. Prosthetic patients	Awareness, Educate, Rights	Treatment option Costs Policy inclusion	Media (print, online); Social media sites Posters, billboards SELF, General practitioners	Frequent reminders	Researcher to share evidence Patients in SDA Clinical Trial and Quality of Life study

### Identifying stakeholders

Several key stakeholders were identified as per their interest related to the SDA or PRDA concept, and they were further grouped for greater impact regarding the communication plan which could ensure implementation of evidence into practice. The stakeholders included lecturers in prosthodontics, senior dental students, dental practitioners (generalist and specialists), dental technicians and other dental auxiliaries, oral health insurers, oral health policymakers, government representatives and most importantly, the dental patients (Fig. 1). Using this strategic mapping approach, these key stakeholders were then grouped, and in no particular order at this stage, according to their specific roles and where they are encountered in the dental environment:

- Academics: These include lecturers in prosthodontics who are expected to reflect on the curriculum and make the necessary changes using evidence-based research, the clinical teachers who are responsible for guiding students to evidence-based practices and researchers whose responsibility include guiding students (under- and post-graduate) and staff to engage with quality research.
- Policymakers: This group would focus on government agents who are responsible for updating oral health policies and oral health insurers who regularly engage practitioners on providing quality care and remuneration policies related to these.
- Practitioners: These are general dentists and specialists, both in the public and private sectors. They are the direct providers of Prosthodontic treatment options and this is where the greatest change should be effected.
- Patients: These are the recipients of these extensive Prosthodontic treatment options and are sometimes not educated about all the options at their disposal, including the SDA or PRDA (Table 1).

### Prioritizing stakeholders

These key stakeholders were grouped so that their impact and influence could be better utilized to guide the researcher with translation of SDA or PRDA research into clinical practice (Tables 1 and 2). Thus, with this stakeholder mapping approach it was possible to highlight the role of each stakeholder that could effect change appropriately and successfully and within the different settings. Moreover, the appropriate stakeholders were also mapped according to their levels of influence and interest related to effecting change with implementation of the SDA or PRDA into clinical practice (Table 2). Stakeholder prioritizing also occurs with this mapping approach and due to this, it is possible to map their level of influence against their measured interests. For example, the level of influence of academics (both lecturers and clinical teachers) in changing the curriculum upon reflection of quality evidence obtained from high-end research and implementing the SDA or PRDA in clinical practice with students, impacts greatly on how they practice as future practitioners, after having received this knowledge. If the curriculum does not address the teaching of the SDA or PRDA using quality evidence, students may continue replacing all missing teeth for all patients after graduating as in the traditional clinical approach.

**Table 2:** Stakeholder interest versus influence in SDA research.

INFLUENCE	HIGH		1. Patients 2. Dental organizations, researchers (evidence/guidelines) 3. Media (print, online, social)	1. Academics (educationalists, clinical teachers, prosthodontists) 2. Practitioners (dentists, prosthodontists), 3. Oral health insurers
	SOME		Oral hygienists	Dental technicians
	LOW	Dental Companies		1.Government policymakers 2. Dental auxiliaries
		LOW	SOME	HIGH
		INTEREST		

Consequently, those with greater influence that may assist with SDA or PRDA implementation such as academics, dentists, and patients must be kept satisfied, managed closely and these stakeholders must be addressed at all times to ensure this. Stakeholders with lower levels of interest must be monitored and kept informed as their role could be seen as supportive (Table 2). The plotting of these stakeholders was completed from the researcher’s perspective stemming from the data gathered while doing this SDA or PRDA research, where the focus was conducting high-end quality research using the evidence pyramid as a guide. The outcomes could possibly look different from another researcher’s or practitioner’s point of view, depending on which aspect of the concept or research they consider more valuable or important. For example, if they are not adamant about changing clinical practice or clinical application of concepts, they might be content with just obtaining a publication (the diffusion and passive component of dissemination of KT).^33^

### Communication plan

By utilizing the stakeholder communication plan, a structured outline of the purpose of engaging them were recorded, how the message will or may be relayed or shared is highlighted, the media or forum to achieve this and the people that could possibly and successfully engage each stakeholder were also noted (Table 1).^39–44^ However, for the SDA or PRDA research completed in the SA context, only some or a few of the stakeholders had been approached at this stage, none of the group of policymakers (government or health insurers) had been engaged yet. Following contextual research, the initial phase of dissemination includes engaging academics and researchers at different fora to highlight the value and major benefits for the SA population regarding the inclusion of the SDA or PRDA treatment approach. At this stage, it would be prudent to delay meeting other stakeholders, for example, clinical practitioners, policymakers, and patients. This will allow reflection on the current approach and consideration regarding the SDA or PRDA option by those approached in the initial phase. This includes observing a subsequent change in their clinical treatment practices, teachings, and patient education. During such a waiting period or following stages of reflection on their current actions after being exposed to quality evidence, it is advisable not to engage other stakeholders as yet.

The next phase included a consultation with the head of the department in Prosthetics, requesting a lecture on the SDA be included in the 4th year of the Dentistry program (Table 1). This was subsequently abandoned as students misunderstood the use of the concept, how to clinically manage these patients and how implementing the SDA in clinics relates to the minimum clinical requirements for the module which forms part of their assessments. This allowed a better understanding of the location of teaching this SDA concept, which was subsequently considered. The idea is to include it in 5th year, as students will be guided to implement it in clinical practice after graduation, and when appropriate. This was an explanation of how one of the influential stakeholders was addressed and the communication plan that was used and how it was modified (Table 1). But with regard the students’ clinical requirements and the inclusion of the SDA and the modifications it will require, there is still a need for it to be addressed on a departmental and faculty level.

With regards to other stakeholders, their purposes, roles, how they were and may be addressed and where these were implemented, if at all, are indicated on the communication plan (Table 1). Different ways of addressing these stakeholders and using different media appropriate to their environment has also been highlighted on this communication plan, for example, researchers would naturally gather at conferences where the evidence was shared with them (Table 1). As stated previously, the communication with different stakeholders cannot ensue concurrently or even chronologically as indicated on the table, but after the initial phase careful planning, reflection and modifications to this plan described, may be considered as and when appropriate.

## Discussion

A stakeholder is an individual or a group of individuals who may be responsible for or who are affected by health-related decisions that must be informed by research evidence. The stakeholders, as relates to the evidence of the SDA or PRDA concept, are all those who are affected by decisions and policies formulated, modified, or implemented as dictated by it. With the stakeholder mapping approach, several key role players were identified, who otherwise would have been overlooked, other than those included in the completed research In South Africa. In addition, their role in the implementation of the SDA or PRDA in clinical practice became more apparent, and what could and should be done to ensure more valuable input and changes to clinical practice that could benefit a large majority of patients.

Knowledge translation (KT) has been described differently by different medical researchers the world-over, and this is indicative of how this aspect of the research and implementation of the evidence has captured researchers.^33–39^ But it seems that researchers are unaware of all the steps involved in achieving a successful outcome following even rigorous research.^33–40^ Most of the researchers, irrespective of the type of research design or procedure or materials researched, manage to present their work at conferences with the intention to publish, but very few follow through on what happens after that. So, up to the stage of dissemination (that is a successful publication), most researchers meet these initial criteria of KT, but few insist on following through on the latter and/or implementation phase of the evidence.^33–39^

Similarly, global evidence related to the SDA concept has been available for many years, yet when clinicians are approached regarding its implementation, they are not convinced of the benefits or of instituting a change in their treatment planning approach. This was seen with the research conducted contextually within SA, as many colleagues said they have never heard about it, with some knowing about it but still did not apply it in clinical practice.^3^ Likewise, studies on the SDA or PRDA from the apex of the evidence pyramid has been conducted globally and now in SA, which implies the quality and value of evidence has increased, yet clinicians hardly offer it as a treatment option to patients where it is clinically appropriate or to those who cannot afford expensive Prosthodontic management options.^5,11–22,32^

With this mapping approach, several stakeholders were highlighted and key professionals who could make a difference to clinically manage patients presenting with SDAs were identified.^34,39–44^ This process also allowed a better understanding of the particular role of key stakeholders who can ensure that this important process of clinical implementation of the SDA becomes a reality.

The impact of changing policy without engaging stakeholders that would be responsible for implementing it, has also been highlighted.^7^ This was seen with the situation in SA, where the oral health policy included the SDA option, but this had not filtered down to the clinical practitioners who treat these patients daily.^7^ More importantly, patients who are direct recipients of treatment related to the SDA should also be engaged and educated in this regard, but this has not happened other than with the studies completed in SA.^5,31^ Naturally, the only stakeholder that was thought of as important initially in this implementation phase, was the clinical practitioners. The question that arises from this is “how can the role of the dental practitioners influence the implementation of the SDA treatment option?”

It has been observed that continuous professional development, which has become compulsory for registered dental professionals in SA, has not been very effective in influencing and changing clinical behaviors and practices. The only other option which will have a direct bearing on the mindset and clinical practices, would be the teachings at the tertiary institutions.^3,4^ To this end, attempts have been made to make a change, but greater action needs to be taken.^4^ Once the teachings are changed, such as in Tanzania, it may influence decision-making among future practitioners.^34,44]^

By completing the stakeholder mapping, the benefits of this process as it relates to key role players was highlighted.^34,39–44^ Researchers are also guided by this stakeholder approach to further identify groups amongst these role players according to their influence and the power they possess to effect change in clinical practice.^34,39–44^ The other advantage of using this mapping approach is to win support of those who can make a meaningful difference as it relates to changing clinical practice, but also use their influence to encourage other colleagues to follow suit (referred to as active support). There are other benefits that have been identified with this type of stakeholder mapping approach and these include:

- The opinions of influential stakeholders may guide your research to success.
- The presence of influential people not only ensures their support, it may also increase the quality of the research being conducted.
- The presence of powerful stakeholders will ensure resources or funding become easily available.^34,39–44^

A stakeholder analysis can be a guide to generate knowledge about people, including understanding their intentions, behavior, interrelations, agendas, interests, and influences.^34,39–44^ By completing the stakeholder communication plan and including how to engage each role player identified, the realization of what resources may be at their disposal (or not) which may influence future decision-making and clinical practices, was highlighted.^34,39–44^ The research related to the SDA or PRDA was important, and more so this mapping process, as the influence of individuals and organizations that are essential in changing clinical practice was emphasized. The role of policymakers is also significant, but the relevant people must be engaged when wanting to ensure a policy will be implemented, otherwise it will be overlooked, as in the case of the SDA or PRDA in SA. Policy formation does not automatically imply implementation, and the role of medical or health insurers to accept or change their payment protocols as it benefits them must also be avoided, as the SDA or PRDA research would like to ensure that neither patients nor practitioners are disadvantaged in any way.

Thus, the discussion related to the SDA or PRDA research, evidence-based practice and changing clinical practice is incomplete without any reference to the financial implications it may have for practitioners not to replace lost molars with either fixed or removable protheses. With the SDA or PRDA research, practitioners who admitted having knowledge about this treatment option were clear about not implementing it as their income would be affected negatively.^3^ Therefore, their actions are influenced by loss of finances, which is a real concern. This sentiment only confirms that there are areas related to the SDA or PRDA treatment approach which is clearly not understood in this process of clinical implementation. First, it has never been said or even implied that all patients must be treated with the SDA treatment option. Moreover, patients with SDAs must be guaranteed of a complete and lengthy follow-up protocol to preserve the teeth present and to ensure success of treatment. This may translate into practitioners not losing income at all.^17^

Using the stakeholder communication plan as a guide, this type of information may then be shared with practitioners to dispel any fears related to income loss. Moreover, the anxiety of income loss may not necessarily be so, and this was demonstrated by the Irish group who explored the economic evaluation of an SDA.^17^ Avoidance of engaging with the financial aspect of SDA research, could therefore have been the reason for the low response rate to the questionnaire sent to SA practitioners a few years ago.^3^ To re-emphasize, set protocols need to be in place and followed when treating a patient with an SDA treatment option, otherwise maintaining this type of occlusal setup will end in failure of treatment. Thus, the conscious delay in changing the clinical requirements in the academic program.

While conducting the SDA research in SA, and engaging with the different cohorts of students, clinical teachers and patients, the benefits of providing the SDA or PRDA treatment option was seen by many. Moreover, clinical decision-making and treatment planning in patients with an SDA or PRDA and who are unable to afford expensive fixed or removable treatment options, would now be based on reliable evidence. Thus, engaging with available evidence ensures empowering clinical practitioners and their practices; this is an approach which cannot be overemphasized. The one place where this could be done without fear or hesitation, would be within the teaching environment.

As mentioned above, SDA research has been conducted for many years and in different settings, and reflecting on the types of evidence published, the reliability of this is unquestionable. But what is it that makes practitioners not act upon and apply this evidence, and more importantly how can we teach clinicians evidence-based practice? From the many learning theories, we know adults are self-directed, motivated, responsible and very practical and problem-centered about new teachings.^39^ Besides what has been mentioned above already, there is no doubt that adults also learn differently, and will respond otherwise to the same teachings. Relating to all the different stakeholders, the message to ensure implementation of the SDA or PRDA clinically should thus be conveyed in a different manner for each of them, as recorded on the communication plan used for this study.^39–42^ Hence, other than the step-by-step approach of KT, other designs to secure application of an intervention clinically may be followed, such as Grol’s sequential approach to implementation which is based on the stages of change theory of clinicians’ behavior.^39–44^ The goal of these different stages is to ensure improved patient care by confirming the implementation of change in clinical practice.

### Limitations

Lack of government involvement in the SA research, even though the SDA concept has been accepted into policy is a major limitation of this research. Being a practitioner, and being cognizant of how the mindset operates related to any changes that will affect day-to-day business, is a limitation in taking this research further. The lack of involvement of oral health insurers in the SDA or PRDA research that could assist in making the SDA treatment option more visible, has also been a limitation. But including them as influential stakeholders while implementing a phased approach using mapping methodology is a step in the right direction.

## Conclusion

This strategic analysis assisted in identifying key stakeholders, and grouping them according to important roles occupied within the dental environment which could assist with implementation of the SDA or PRDA concept. The role of these stakeholders should be addressed further to ensure alignment to SA oral health policy, but more importantly, engaging with each of them is also crucial to allay unspoken fears and misunderstandings.
